# Hydroxyurea treatment is associated with reduced degree of oxidative perturbation in children and adolescents with sickle cell anemia

**DOI:** 10.1038/s41598-020-76075-5

**Published:** 2020-11-04

**Authors:** Caian L. Vinhaes, Rozana S. Teixeira, Jay A. S. Monteiro-Júnior, Rafael Tibúrcio, Juan M. Cubillos-Angulo, María B. Arriaga, Adrielle G. Sabarin, Amâncio J. de Souza, Jacqueline J. Silva, Isa M. Lyra, Ana Marice Ladeia, Bruno B. Andrade

**Affiliations:** 1 Instituto Gonçalo Moniz, Fundação Oswaldo Cruz (FIOCRUZ), Salvador, 40296-710 Brazil; 2 Multinational Organization Network Sponsoring Translational and Epidemiological Research (MONSTER) Initiative, Salvador, 41810-710 Brazil; 3 School of Medicine, Faculdade de Tecnologia E Ciências (UniFTC), Salvador, 41741-590 Brazil; 4 Bahiana School of Medicine and Public Health, Bahia Foundation for the Development of Sciences, Salvador, 40290-000 Brazil; 5 School of Medicine, Federal University of Bahia, Salvador, 40110-100 Brazil; 6 University Salvador (UNIFACS), Laureate International Universities, Salvador, 41720-200 Brazil; 7 Catholic University of Salvador, Salvador, 41740-090 Brazil

**Keywords:** Biomarkers, Haematological diseases

## Abstract

Sickle cell anemia (SCA) is the most common inherited hemolytic anemia worldwide. Here, we performed an exploratory study to investigate the systemic oxidative stress in children and adolescents with SCA. Additionally, we evaluated the potential impact of hydroxyurea therapy on the status of oxidative stress in a case–control study from Brazil. To do so, a panel containing 9 oxidative stress markers was measured in plasma samples from a cohort of 47 SCA cases and 40 healthy children and adolescents. Among the SCA patients, 42.5% were undertaking hydroxyurea. Multidimensional analysis was employed to describe disease phenotypes. Our results demonstrated that SCA is associated with increased levels of oxidative stress markers, suggesting the existence of an unbalanced inflammatory response in peripheral blood. Subsequent analyses revealed that hydroxyurea therapy was associated with diminished oxidative imbalance in SCA patients. Our findings reinforce the idea that SCA is associated with a substantial dysregulation of oxidative responses which may be dampened by treatment with hydroxyurea. If validated by larger prospective studies, our observations argue that reduction of oxidative stress may be a main mechanism through which hydroxyurea therapy attenuates the tissue damage and can contribute to improved clinical outcomes in SCA.

## Introduction

Sickle cell anemia (SCA) is the most common monogenic hemoglobinopathy disease in the world^1,2^. This disease is characterized by altered hemoglobin synthesis (sickle hemoglobin [HbS]), which leads to several pathological effects, including hemolysis, vaso-occlusive crises, progressive organic damage, and eventual early death^3^. Notably, such hereditary hemolytic anemia exhibits a high prevalence in Brazil, especially in the state of Bahia^4^. Due to the scarcity of an effective pharmacological treatment, understanding of fundamental mechanisms underlying SCA may lead to development of novel therapies and optimized patient care^1,5,6^.

Numerous aspects are long proposed to influence the pathogenesis of SCA^7,8^. Notably, a large number of studies investigating oxidative stress in SCA patients have indicated that several types of reactive oxygen species (ROS) affect red blood cells (RBCs), resulting in metabolic dysfunction of these cells and alteration of their biochemical properties^9,10^. There is also evidence supporting the idea that oxidative damage mediates cytoskeleton remodeling in RBCs, leading to morphological deformities, and consequently affecting traffic through smaller capillaries. Many consequences of this process have been described, such as inflammatory endothelial injury, platelet adhesion, vaso-occlusion, nitric oxide depletion, ischemia and pain^1,11,12^.

The clinical management of SCA patients is a largely explored field with several lines of investigations^12–14^. Among the treatment options, the use of hydroxyurea has emerged as an important pharmacological intervention^1,15,16^. Nevertheless, although hydroxyurea is now largely used in SCA to prevent acute vaso-occlusive crises, the specific effects of hydroxyurea therapy on the oxidative response remains uncertain. Here, we performed an exploratory case–control study in Brazilian children and adolescents using multidimensional analysis to evaluate the expression of biomarkers related to oxidative stress, measuring the global oxidative perturbation in SCA and comparing to that detected in non-SCA healthy controls. In addition, we evaluated whether hydroxyurea treatment is associated with changes in such oxidative perturbation in SCA patients.

## Materials and methods

### Ethics statement

All clinical investigations were conducted according to the principles of the Declaration of Helsinki. Written informed consent was obtained from each participant or legal guardian at the study enrollment. This study was approved by the Ethics Committee of the Bahiana School of Medicine and Public Health, (protocol number: 568.913/2014; C.A.E.E. no. 17663913.2.0000 5544). Access to the registry data was authorized by the boards of the participating institutions.

### Study design and participants

In the present study, plasma samples were collected from 47 children and adolescents from 6 to 18 years old, diagnosed with SS Hemoglobinopathy, enrolled and followed up at the Hematology Outpatient Clinic of the Magalhães Neto Ambulatory—Professor Edgard Santos University Hospital Complex (HUPES) of the Bahia School of Medicine—UFBA and at the Foundation for Hematology and Hemotherapy of Bahia (HEMOBA). The aforementioned institutions are reference centers in the care and treatment of hematological diseases in the state of Bahia, Brazil. Inclusion criteria for sickle cell patient recruitment included HbSS diagnosis via electrophorese or high-performance liquid chromatography (HPLC), and absence of recent infectious or SCA-related acute events. The current study was a case–control analytical investigation with cases (SCA patients) and control group (non SCA individuals), performed between March 2013 and June 2015. Additional comparisons within the SCA group were performed between participants stratified based on use of hydroxyurea. Plasma sample collection was performed from June 1, 2014 to March 31, 2015. Healthy participants consisted of children and adolescents of the same age group, whose inclusion criteria included absence of sickle cell disease determined by Hb electrophoresis and/or high-performance liquid chromatography, or any other acute or chronic diseases, enrolled at the general pediatric teaching-care ambulatory of Roberto Santos General Hospital (HGRS) / Bahiana School of Medicine and Public Health (EBMS) and at the outpatient clinic of Hebiatra of the Magalhães Neto Ambulatory—HUPES Complex of the Bahia Medical School—Federal University of Bahia (UFBA).

### Laboratory measurements

Blood samples were obtained after a fasting period of at least 8 h^17,18^. The following parameters were assessed in a reference laboratory: hematocrit (%), hemoglobin (g/dL), mean corpuscular volume (MCV) (fL), corpuscular hemoglobin concentration (MCHC) (pg), total leukocyte count (× 10^9^/L), platelet count (× 10^9^/L), reticulocyte count (%), Glutamic oxaloacetic transaminase (GOT) (U/L), glutamate-pyruvate transaminase (GPT) (U/L), direct bilirubin (DB) (mg/dL), indirect bilirubin (IB) (mg/dL), total cholesterol (mg/dL), low density lipoprotein-cholesterol (LDL-c) (mg/dL), high density lipoprotein-cholesterol (HDL-c) (mg/dL), triglycerides (mg/dL), and high sensitivity C-reactive protein (CRP) (mg/L), as previously described^17,18^. Details of the assays described below, which used to assess oxidative stress, have been formerly published^19^. Total oxidant status was assessed using a commercially available kit (Rel Assay Diagnostics, Gaziantep, Turkey) and results are described as micro molar hydrogen peroxide equivalent per liter (μmol H_2_O_2_ Equiv./L). Total antioxidant status was measured using the Antioxidant Assay kit from Cayman Chemical (Ann Harbor, MI), where the capacity of the antioxidants to counteract ABTS (2,2′-azino-di-[3-ethylbenzthiazoline sulphonate]) oxidation is compared with that of Trolox, and is calculated as molar Trolox equivalents^19^. Lipid peroxidation in plasma was quantified using a kit from Cayman Chemical, which measures the formation of malondialdehyde (MDA)^19^. Levels of lactate dehydrogenase (LDH), glutathione (GSH) and of superoxide dismutase protein level (SOD) were measured using kits from Cayman Chemical following the manufacturer’s protocol. ELISA commercial kits from R&D Systems (Minneapolis, MN) were used to quantify concentrations of soluble CD14 (sCD14), heme oxygenase-1 (HO-1) and of vascular endothelial growth factor (VEGF).

### Data analysis

Descriptive statistics were performed to characterize the study population. Continuous variables were tested for Gaussian distribution using the D’Agostino-Pearson test. For parameters which values exhibited a non-Gaussian distribution, the median values with interquartile ranges (IQR) were used as measures of central tendency and dispersion. The Mann–Whitney *U* test (when 2 groups were compared) or the Kruskal–Wallis test with the Dunn’s multiple-comparison (when more than 2 groups were compared) were used to evaluate continuous variables. For variables which values displayed a Gaussian distribution in the study population, Student’s t test (for 2 groups) or One-Way ANOVA with the Tukey’s ad hoc were employed to compare the groups. The Fisher’s exact test was used to compare variables displayed as frequencies (%). We used principal component analysis (PCA) to test which combination of oxidative stress related markers could distinguish individuals with or without SCA. The PCA integrated with vector analysis was chosen due to its capacity to perform dimension reduction of multiple biomarker measurements and display the overall distribution of the global data variation. This approach also allowed us to visualize the impact of each parameter inputted on the distribution of the data and individuals from the distinct clinical groups. A hierarchical cluster analysis (Ward’s method) of log10 transformed and z-score normalized data was employed to depict the overall expression profile of indicated biomarkers in the study subgroups, as described previously^20–22^. All comparisons were pre-specified and two-tailed. Statistically significant differences were defined as displaying p-values < 0.05 after Holm-Bonferroni’s adjustment for multiple comparisons. Profiles of correlation between oxidative stress parameters were examined using network analysis of the Spearman correlation matrices as previously described^23,24^. Correlations with p-value < 0.05 were included in the network visualization.

The molecular degree of perturbation was calculated using values of the markers related to oxidative stress to infer the degree of oxidative perturbation (DOP) associated with SCA and impacted by hydroxyurea therapy. This method has been used and detailed previously^20–22,25,26^. In the present study, “healthy control” (non-SCA) was defined as the “reference” group and the average level and standard deviation of this reference group was calculated for the plasma concentrations of each plasma marker^20–22,25,26^. The DOP score of an individual marker in a given sample “s” was defined by taking the difference in concentration level in sample “s” from the average of the marker in reference group divided by the corresponding standard deviation^20–22,25,26^. Thus, the DOP score represents the number of standard deviations from the reference^20–22,25,26^.

## Results

### Characteristics of the study population

A total of 87 children and adolescents were enrolled, 47 SCA patients (HbSS confirmed), from whom 20 were undertaking hydroxyurea, and 40 healthy controls (Table S1). Participants were similar with regard to age (SCA median [IQR] 13 years [9–16] vs. healthy control median [IQR] 11 years [8–16]; P = 0.14) (Table S1). Male sex predominated in the group of SCA (57.4%; n = 27), whereas female sex was more frequent in the healthy control group (67.5%; n = 27) (P = 0.03) (Table S1). The study population was predominantly composed by non-white individuals independent of the study group, corresponding to 95.7% (n = 45) of the SCA group, and 82.5% (n = 33) among healthy persons (P = 0.13) (Table S1).

### Clinical and laboratorial evaluation between the clinical groups

Laboratory and anthropometric parameters were assessed and compared between the study groups (Table 1). As expected, values of oxygen saturation, hemoglobin and hematocrit were higher in individuals from the healthy control group (Table 1). Of note, levels of total cholesterol, LDL-c and HDL-c were also higher in those without SCA (Table 1), whereas leukocyte count, circulating levels of aspartate aminotransferase (AST), alanine aminotransferase (ALT), triglycerides and CRP were higher among SCA patients who were not undertaking hydroxyurea therapy (Table 1). In addition, SCA patients that were being treated with hydroxyurea exhibited higher values of mean corpuscular volume (MCV), platelet count, total bilirubin and indirect bilirubin. Importantly, extending the analysis to directly compare the subgroups of SCA patients based on hydroxyurea treatment using the Mann–Whitney *U* test revealed that only two parameters (MCV and direct bilirubin) were statistically significant, with higher values being detected in participants treated with hydroxyurea (Table 1). These findings confirmed that the SCA patients indeed exhibit indication of red blood cell lysis and activation of compensatory mechanisms and suggest that hydroxyurea therapy may be associated with attenuation of the hemoglobin polymerization.

**Table 1:** Characteristics of the clinical groups.

Parameter	Unit	Healthy control (n = 40)	SCA No HU (n = 27)	SCA HU (n = 20)	*P*-value
SpO_2_	%	98 (97–98)	93.5 (88.2–96.0)	94.5 (93.0–97.0)	**0.001**
BMI	Kg/m^2^	17.2 (14.8–20.2)	17.3 (15.1–19.0)	16.1 (14.6–17.6)	0.34
Hemoglobin	mg/dL	12.4 (11.7–14.2)	8.0 (7.5–8.5)	8.0 (6.9–8.5)	**< 0.001**
Hematocrit	%	38.8 (35.5–41.7)	22.6 (21.7–25.0)	23.9 (22.0–27.0)	**< 0.001**
MCV	fL	84.3 (78.2–89.4)	88.8 (80.1–92.4)	96.9 (90.5–101.4)	**0.005**^**a**^
MCHC	pg	33.1 (32.0–34.2)	33.8 (30.9–35.5)	33.9 (33.3–35.5)	0.24
Leukocytes	10^9^/L	7.0 (6.2–8.0)	13.0 (10.3–14.9)	11.5 (9.6–15.4)	**< 0.001**
Platelet	10^9^/L	284.0 (248.5–338.3)	440.0 (339.5–531.5)	494.0 (444.0–582.0)	**0.001**
Reticulocytes	%	0.8 (0.7–6.1)	5.9 (3.2–9.1)	5.9 (2.8–11.0)	**0.02**
AST	U/L	22.0 (15.0–29.5)	52.0 (40.6–69.1)	48.0 (29.3–68.0)	**< 0.001**
ALT	U/L	15.0 (12.0–18.0)	24.5 (16.5–34.3)	21.0 (15.5–35.5)	**0.01**
Total bilirubin	mg/dL	0.48 (0.22–0.83)	2.8 (2.4–5.0)	3.6 (1.6–3.8)	**< 0.001**
Direct bilirubin	mg/dL	0.08 (0.04–0.24)	0.64 (0.5–0.93)	0.32 (0.28–0.5)	**< 0.001**^**a**^
Indirect bilirubin	mg/dL	0.35 (0.2–0.5)	2.0 (1.6–4.5)	3.1 (1.4–3.5)	**< 0.001**
Total cholesterol	mg/dL	149.0 (128.1–174.8)	123.5 (105.9–135.8)	109.5 (97.4–129.0)	**< 0.001**
LDL-c	mg/dL	83.2 (74.1–92.4)	73.1 (54.0–84.3)	60.6 (50.9–69.0)	**< 0.001**
HDL-c	mg/dL	43.3 (37.7–56.1)	30.4 (22.2–36.0)	30.2 (28.3–36.9)	**< 0.001**
Triglycerides	mg/dL	67.8 (49.7–85.9)	101.6 (86.1–139.2)	94.5 (73.8–115.6)	**< 0.001**
CRP	mg/L	0.8 (0.22–2.0)	2.2 (0.9–3.7)	2.0 (1.1–8.4)	**0.001**

Data represents median and interquartile range (IQR) and were compared using the Kruskal–Wallis test.

^a^Markers presented significant P-value in the Mann–Whitney U test between SCA with and without hydroxyurea therapy. (appear order) *SpO2* Oxygen saturation, *BMI* Body Mass Index, *MCV* Mean Corpuscular Volume, *MCHC* Corpuscular hemoglobin concentration, *AST* Aspartate aminotransferase, *ALT* Alanine aminotransferase, *LDL-c* Low density lipoprotein-cholesterol, *HDL-c* High density lipoprotein-cholesterol, *CRP* C Reactive Protein, *HO-1* Heme oxygenase-1, *SOD* Superoxide Dismutase, *GSH* Glutathione, *MDA* Malondialdehyde, *LDH* Lactate dehydrogenase, *VEGF* Vascular endothelial growth factor, *sCD14* Soluble CD14.

### Sickle cell anemia is hallmarked by intense oxidative stress in peripheral blood

**Figure 1:**
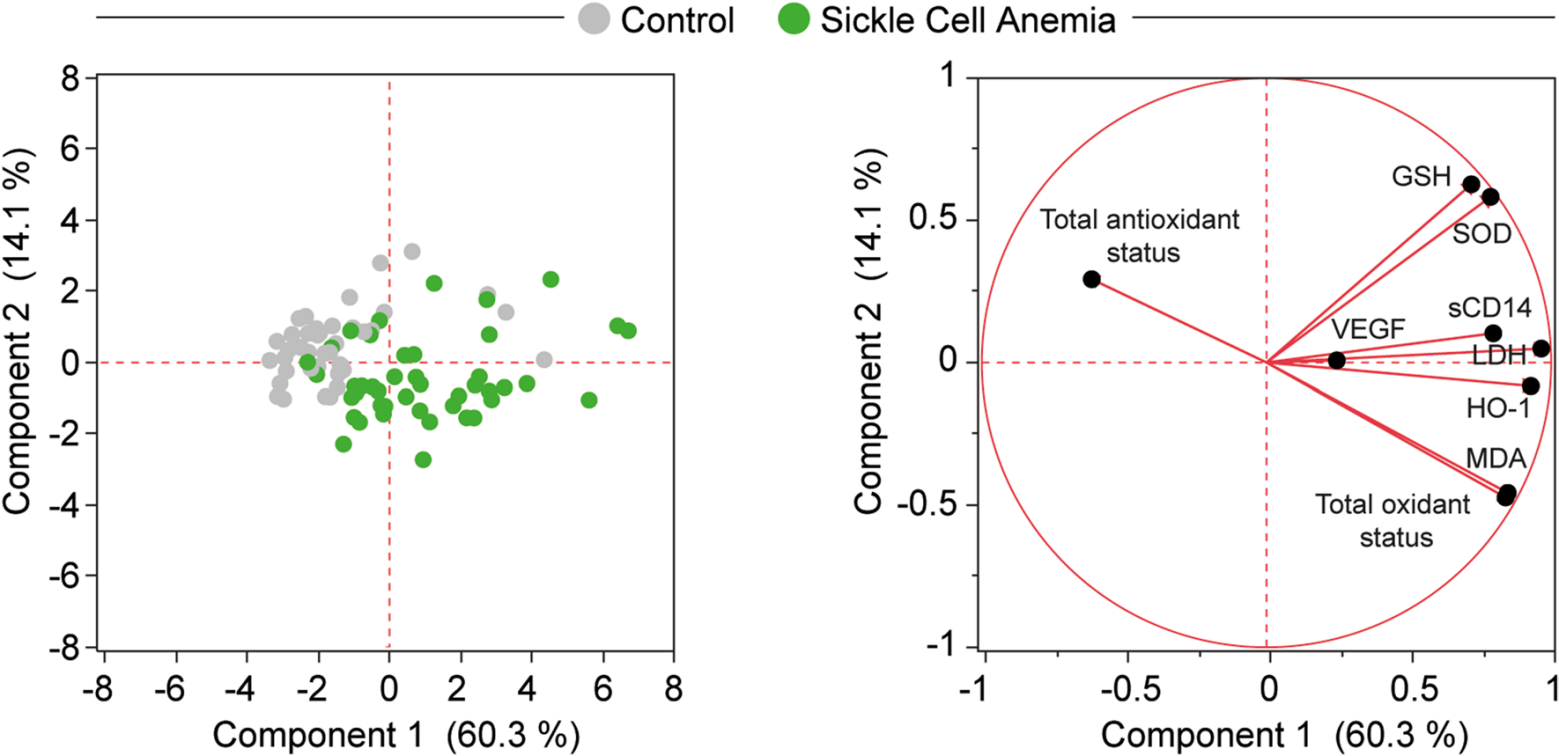
Principal component analysis using oxidative stress related markers can distinguish SCA patients from the healthy controls. A PCA model was employed to test whether combination of the markers evaluated could cluster SCA patients separately from controls. A vector analysis (biplot rays) was utilized to illustrate the influence of each biochemical parameter in the distribution of the data of the PCA model**.**

We extended the analyses to compare the activation profile of pro-oxidative responses in plasma between the study groups. Higher levels of HO-1, total oxidative status, lipid peroxidation (MDA), cell lysis/death (LDH) and sCD14 were found in SCA patients compared to those detected in non-SCA controls (Table S2). Next, we employed a Principal Component (PCA) algorithm with vectors (biplot rays) inputting values of all the oxidative markers measured to test whether the major groups (SCA vs. non-SCA) could be distinguished, and which variables would influence such distinction (Fig. 1).

The vector analysis integrated with a PCA model showed that heightened levels of total oxidant status, MDA, HO-1, LDH, SOD and GSH were more associated with SCA participants, whereas the values of total antioxidant status were associated with the healthy control group. This finding highlights a potential role of oxidative stress in the SCA pathophysiology, with a higher expression of oxidant factors being able to distinguish SCA patients from non-SCA controls.

### Hydroxyurea therapy is associated with reduced oxidative stress in sickle cell anemia patients

**Figure 2:**
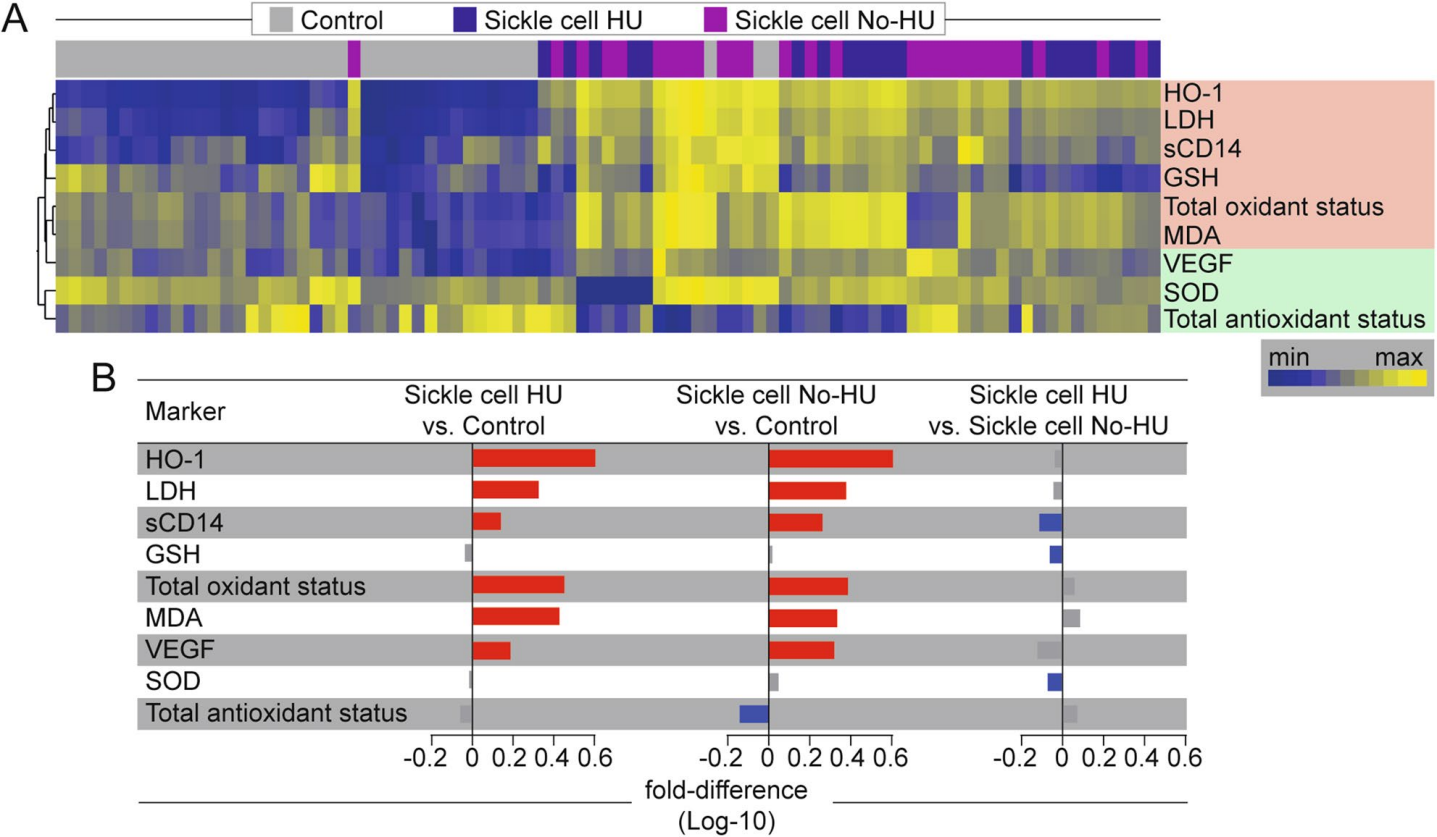
Sickle cell anemia is linked to a distinct profile of oxidative stress. (**A**) An unsupervised, two-way hierarchical cluster analysis (Ward’s method with 100 × bootstrap) was employed to depict the overall expression of plasma markers associated with oxidative stress in study population. Two major clusters of markers were observed and are highlighted in red and green boxes on the right. (**B**) Average fold-difference values in plasma markers for subgroups of SCA participants based on use of hydroxyurea therapy and healthy controls. Differences that reached statistical significance (Mann–Whitney *U* test) after adjustment for multiple comparisons (adjusted P-value < 0.05) are represented in color bars. Red bars indicate higher values whereas blue bars denote lower values in the comparison group as indicated. (Order of appearance) *HO-1* Heme oxygenase-1, *HU* hydroxyurea, *LDH* Lactate dehydrogenase, *sCD14* Soluble CD14, *GSH* Glutathione, *MDA* Malondialdehyde, *VEGF* Vascular endothelial growth factor, *SOD* Superoxide Dismutase.

Given that results reported above indicated that SCA is hallmarked by increased oxidative stress, we further investigated the possible impact of the hydroxyurea therapy in this oxidative milieu. To do so, we compared the expression of oxidative markers between subgroups of SCA patients stratified according to use of hydroxyurea (Fig. 2).

An unsupervised two-way hierarchical clustering analysis of the log10-transformed and z-score normalized values/concentrations of each parameter uncovered a clear distinction of oxidative profiles between the SCA and controls when all markers were examined simultaneously, revealing a lower expression of the all oxidative markers in healthy controls, except for the total antioxidant status (Fig. 2A). This finding argue that the oxidative stress profile is very distinct in SCA patients. This analysis also indicated that SCA patients undertaking hydroxyurea therapy could not be grouped separately from those not using such treatment (Fig. 2A). With regard to the markers evaluated, two main clusters were observed. The first cluster (shown in red in Fig. 2A) included HO-1, LDH, sCD14, GSH, total oxidant status and MDA, whereas the second cluster (shown in green) included VEGF, SOD and total antioxidant status. To quantify differences in expression of the oxidative markers between the study groups, we employed a fold-difference analysis (Fig. 2B) and found higher levels of HO-1, LDH, sCD14, total oxidant status, MDA and VEGF in SCA participants who were undertaking hydroxyurea compared to controls (Fig. 2B). A similar tendency was observed in SCA patients who were not taking hydroxyurea when also compared with the control group (Fig. 2B). Of note, in this latter specific comparison, total antioxidant status now showed to be substantially lowered in SCA compared to controls (Fig. 2B), highlighting the skewed systemic response towards pro-oxidation. Interestingly, among the SCA participants, those that were being treated with hydroxyurea displayed significantly lower values of sCD14, GSH and SOD when compared those who were not undertaking such therapy (Fig. 2B). These observations identified an oxidative signature of SCA marked by higher values of HO-1, LDH, sCD14, total oxidant status, MDA and VEGF. The results also argue that SCA is indeed associated with exacerbated accumulation of free radicals that define oxidative stress and that hydroxyurea therapy is associated with reduced systemic oxidative responses.

**Figure 3:**
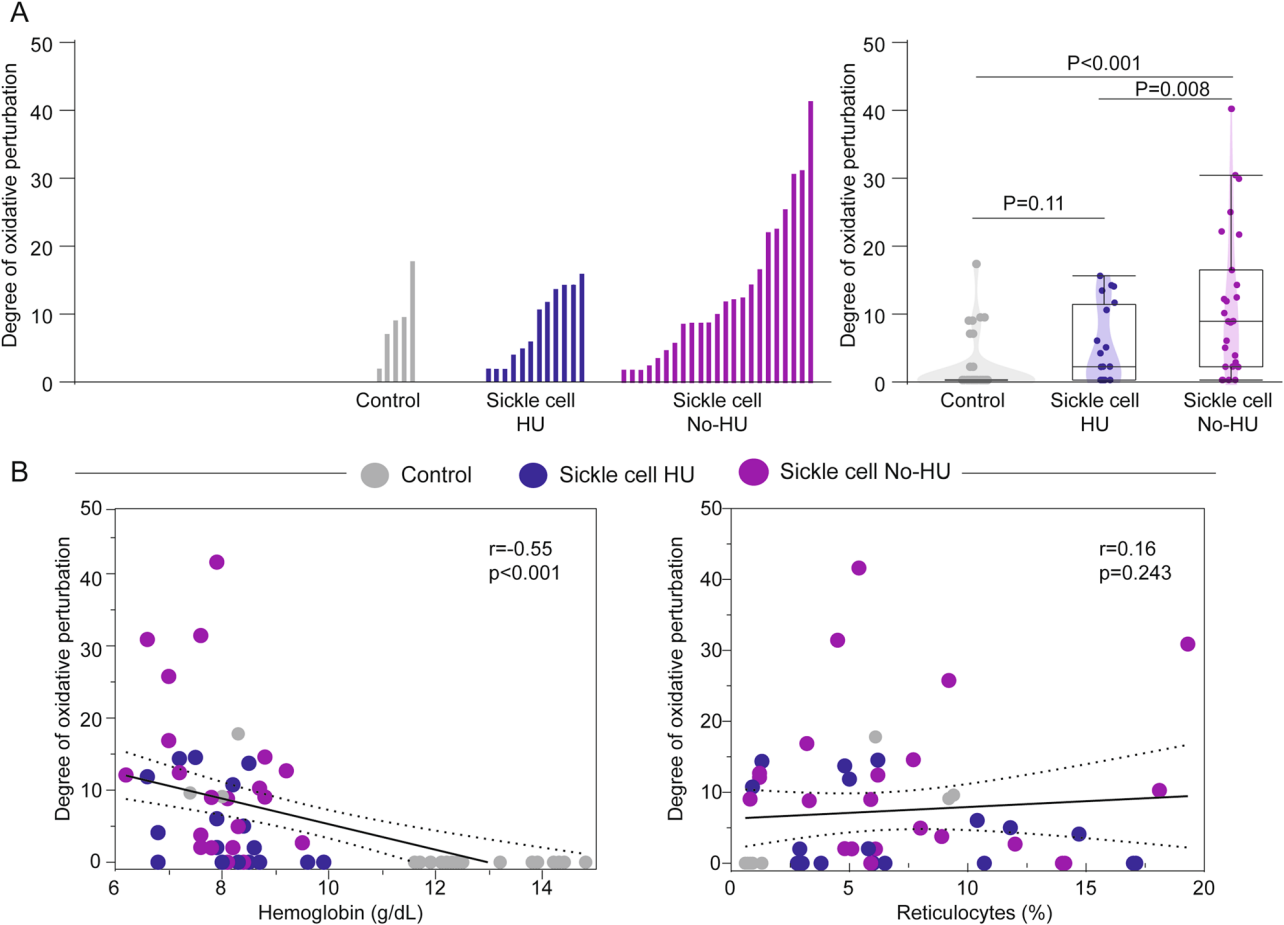
Hydroxyurea treatment is associated with reduced degree of oxidative perturbation in Sickle Cell Anemia. (**A**) (Left panel) Histograms show the single sampe degree of oxidative perturbation (DOP) according the clinical group or helthy controls as indacted. (Right panel) Violin box plots represent the distribution of the DOP score values between study groups. Values were compared using the One-Way ANOVA test with Tukey’s post test, after observing a Gausian distribution of values using the D’Agostino-Pearson test (P-value < 0.001). (**B**) Speraman correlation analysis was performed between the values of DOP and of indicated markers. *HU* hydroxyurea.

To further test the hypothesis that therapy with hydroxyurea could be tightly associated to an attenuated oxidative milieu in SCA participants, we calculated the degree of oxidative perturbation (DOP) (as described in the “Materials and methods” section) (Fig. 3), which is an adaptation from the molecular degree of perturbation previously published^21,25,27^. We also evaluated the correlation between DOP values and hemoglobin levels or reticulocyte count, which are markes directed associated with degree of SCA disease activity (Fig. 3). This novel statistical approach uncovered that the group of patients with SCA who were not undertaking hydroxyurea exhibited the highest values of DOP among the clinical groups (Fig. 3A). Importantly, this analysis also revealed that DOP values were reduced in SCA participants undertaking hydroxyurea therapy, with no clear distinction from values calculated for the non-SCA controls (Fig. 3A). Additionally, the Spearman correlation analyses showed a negative relationship between the values of DOP and hemoglobin levels (r = -0.55; P < 0.001) and a positive, but not statistically significant, correlation between DOP values and reticulocyte count (Fig. 3B). This finding made us hypothesize that higher degree of oxidative perturbation was associated with lower hemoglobin levels, and that hydroxyurea therapy in SCA may lead to consistent decreases in such systemic oxidative perturbation.

### The dynamic of oxidative markers in sickle cell anemia

**Figure 4:**
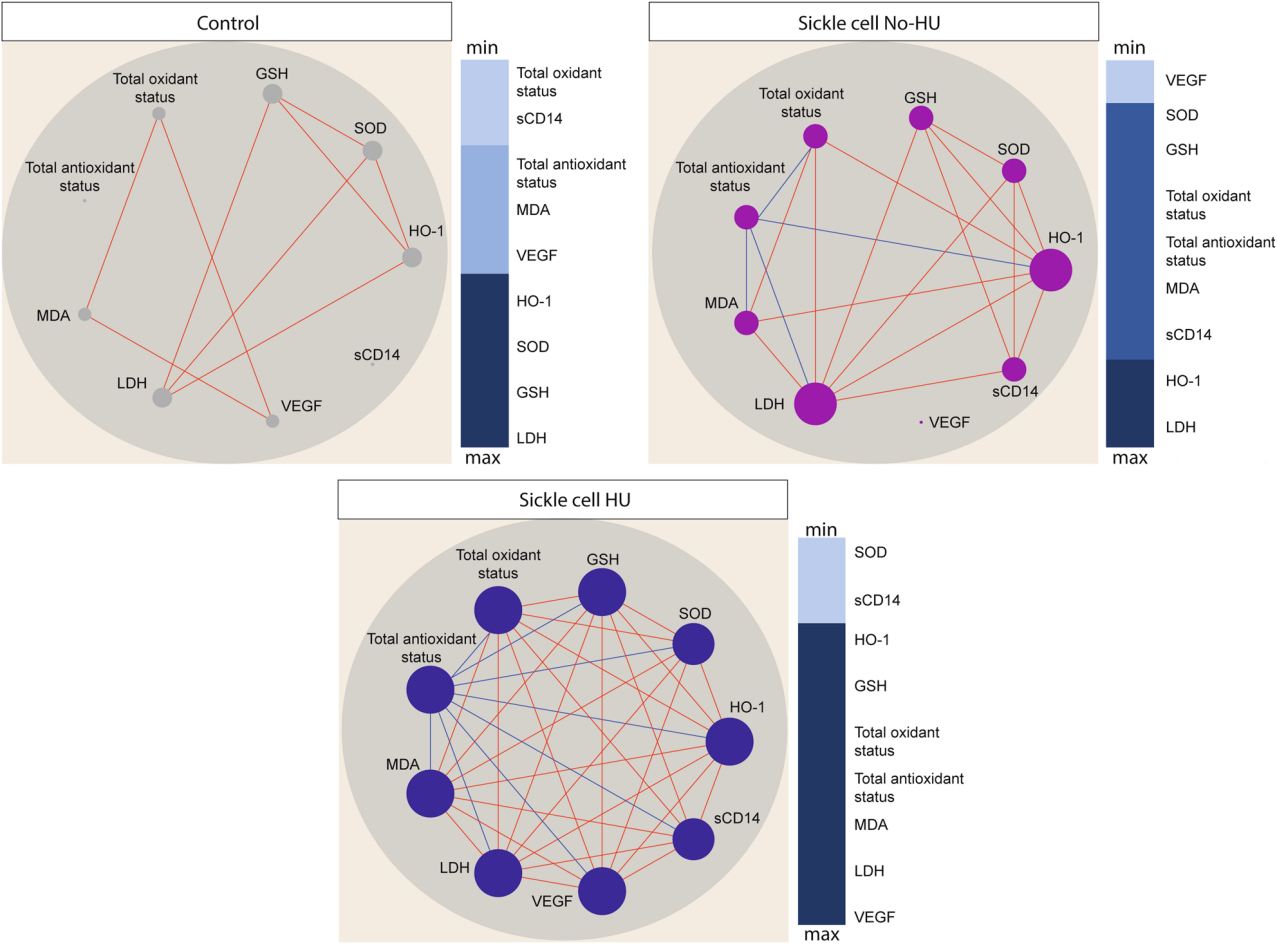
Hydroxyurea therapy is associated with coordinated changes in relationships between oxidative stress markers in SCA. Network analysis of the biomarker correlation matrices was performed with bootstrap (100 ×). The graphs show significant correlations (P < 0.05), and the Spearman rank (rho) threshold was ± 0.5. Each node represents a different parameter. The size of each circle (node) is proportional to the number of significant correlations involving such node. Connecting lines represent the Spearman rank coefficient (rho) values. Red color infers positive correlation, whereas blue color denotes negative correlations. Color maps on the right of each network denote the number of significant correlations per parameter (node) per clinical group as indicated. Heatmaps on the right panels rank the markares in each network based on number of statistically significant correlations involving each individual marker. *HO-1* Heme oxygenase-1, *HU* hydroxyurea, *LDH* Lactate dehydrogenase, *sCD14* Soluble CD14, *GSH* Glutathione, *MDA* Malondialdehyde, *VEGF* Vascular endothelial growth factor, *SOD* Superoxide Dismutase.

The results described above revealed a marked oxidative disturbance in peripheral blood of SCA, and mainly in patients not undertaking hydroxyurea. To depict the nuances of a potential regulation of this pro-oxidative environment, our next exploratory analysis employed a method based on Spearman network correlation, as previously reported^22,23,28^. This statistical approach helped us to identify the interaction, strength and quality of the relationships between values of the markers evaluated in the different study groups. We found that networks of the distinct clinical groups displayed differences in density and quality (e.g. positive vs. negative correlations) of statistical interactions between values of the oxidative stress related markers (Fig. 4). Regardless of the clinical group, most of the statistically relevant correlations were positive, meaning that the higher levels of a given marker were followed by heightened expression of other oxidative parameters. Importantly, the network density (which infers the total number of statistically significant correlations) was the highest in SCA patients that received hydroxyurea therapy, and the lowest in the control group (Fig. 4).

In the healthy control group, only positive interactions were found, whereas among the SCA participants not undertaking hydroxyurea, we observed four negative significant correlations. Such correlations included the total antioxidant status and four other oxidant markers: (i) total oxidant status, (ii) HO-1, (iii) MDA and (iv) LDH (Fig. 4). Of note, while extending this observation to the group of SCA participants undertaking hydroxyurea, we found a higher number of negative correlations, also led by the total antioxidant status, but now interacting with all the other markers (Fig. 4).

The network analysis highlighted that in healthy controls, LDH was the most highly connected marker, followed by GSH, SOD and HO-1 (Fig. 4). In SCA participants not undertaking hydroxyurea, LDH was the most relevant marker with positive correlations with sCD14, HO-1, SOD, GSH, total oxidant status and MDA, and a unique negative correlation with total antioxidant status, as mentioned above (Fig. 4). Importantly, in the SCA patients undergoing hydroxyurea treatment, all markers exhibited interactions each other, except for sCD14 and SOD (Fig. 4). The network from this latter clinical group displayed VEGF, LDH, MDA, total oxidant and antioxidant status, GSH and HO-1 as top highly connected markers (Fig. 3). Thus, we hypothesized that the hydroxyurea therapy may lead to decrease in the oxidative milieu through a coordinated mechanism that may regulate the inflammatory process and that could be visualized through network analysis.

## Discussion

It has been long proposed that oxidative stress plays a critical role in the pathophysiology of SCA^2,29,30^. The identification of oxidative stress related biomarkers is of utmost importance to help understanding the mechanisms that drive the physiopathology and clinical outcomes in SCA^31,32^. Additionally, the paucity of approved pharmacological treatments for SCA hinders the achievement of a highly successful therapeutic intervention as well as appropriate patient care, culminating in poor outcome in carriers of this genetic condition. In the present investigation, we performed a detailed analysis of plasma biomarkers related to redox status and inflammation in SCA children and adolescents undertaking or not hydroxyurea treatment in order to better understand the nuances of the association between oxidative stress and inflammation in this disease. We also tested association between the hydroxyurea therapy and alteration of the oxidative responses in the patients evaluated.

Oxidative stress is a direct consequence of the imbalance between production of free radicals versus total antioxidant factors. It is already known that excessive oxidative responses can culminate in tissue damage and consequently poor outcomes in several pathological conditions. This process has been largely described by our group in a large number of diseases^33–35^. The results presented here expand the disease repertoire linked to oxidative stress as they demonstrated a marked increased oxidative milieu in patients with SCA, which is characterized by augmented total oxidant status as well as increased lipid peroxidation (measured here through of MDA values) and cell lysis/necrosis (read by LDH levels). In addition, higher concentrations of sCD14 and HO-1 were observed in SCA patients. All of those markers have been previously reported individually, but not together, to be associated with SCA and vascular injury^2,18,36,37^. Importantly, the hierarchical cluster analysis inputting values of all the biomarkers measured here indicated that the homeostasis is so dramatically altered by SCA that the combination of such markers could accurately distinguish the patients from the non-SCA control group. The same analysis failed to reliably discriminate the groups of SCA patients undertaking or not hydroxyurea. These findings demonstrate that hyperexpression of oxidative factors in response to SCA hallmark this condition, regardless of the hydroxyurea treatment. This result increments the current knowledge in the field, and identifies parameters involved in the pathophysiology of SCA that may be targeted in future studies exploring individualized therapeutic strategies, as previously suggested^10^.

An important contribution of our study was the assessment of the degree of oxidative perturbation (DOP). To our knowledge, no previous study has estimated the global oxidative disturbance in SCA patients. It is known that the oxidative milieu with production of free radicals would favor hemoglobin polymerization and RBCs sickling, leading to onset of SCA-related clinical manifestations, such as intravascular hemolysis^38^, vessel occlusive syndrome^10,39^. The same pathological process results in complications related to unfavorable outcomes, for example endothelial dysfunction^40^, chronic inflammation^41^, acute chest syndrome^39,42^ and others^43–45^. Despite the recent advances in studies depicting pathophysiologic aspects of SCA, establishment of clinical interventions to avoid SCA-associated complications remains elusive, and tools to infer disease activity are needed. Here, we found an inverse correlation between the DOP levels and hemoglobin, suggesting that the intensity of the oxidative milieu is associated with increased red blood cell degradation and its consequences, or that an intense red blood cell destruction and the consequent tissue damage is associated with increases in the oxidative status. Regardless of the direction of the association, the degree of oxidative perturbation based on the markers explored here hallmarks SCA. Quantifying the oxidative disturbance through DOP could emerge as a useful tool to follow up SCA carriers and guide onset of early interventions. Future prospective studies evaluating more markers and performing mechanistic investigations ex vivo are warranted to directly test the utility of this approach.

A relevant aspect shown here was the decrease of the degree of oxidative perturbation in SCA patients undertaking hydroxyurea. The risks and benefits of this pharmacological intervention are discussed elsewhere^46^, and its effects on chronic complications of SCA remain unclear^1,47^. Nevertheless, hydroxyurea therapy has been associated with decreases in frequency of painful crises^48^, and other complications such as acute chest syndrome, dactylitis, hospitalization and transfusions^1,49^. It has been recently suggested that hydroxyurea therapy could modulate oxidative stress through its effect on the hemoglobin^50^. Here, using the DOP analysis, we found that the hydroxyurea therapy leads to consistent decreases in the overall degree of oxidative perturbation, which values were indistinguishable from non-SCA healthy controls. It is reasonable to hypothesize that hydroxyurea therapy attenuates the oxidative stress status in SCA patients, contributing to clinical improvement and a tendency towards a milder disease. Importantly, the networks of correlation matrices demonstrated an increase in the number of connections and an apparent balance in the oxidative responses, reflected by the similar number of correlations between the markers, in hydroxyurea treated SCA patients. This observation suggest that hydroxyurea therapy may reduce the activation of mechanisms leading to production of free radicals in a coordinated manner, reflected by increased number of statistically significant correlations between concentrations of parameters associated with regulation of oxidative and inflammatory responses. Recently, in vitro analyses using human peripheral blood cell and human umbilical vein endothelial cells treated with hemin demonstrated that hydroxyurea therapy scavenges free radicals and induces the expression of antioxidants genes in these cells^51^, reinforcing our hypothesis. Additionally, we found higher levels of mean corpuscular volume in SCA patients who were being treated with hydroxyurea, which may suggest attenuated polymerization of hemoglobin. Altogether, these observations support the idea that hydroxyurea therapy may mitigate the systemic oxidative stress, directly scavenging free radicals and indirectly inducing transcription of antioxidant genes, contributing to reduce the hemoglobin polymerization and, consequently, all unfavorable clinical events linked to the cell sickling, such as painful crises, acute chest syndrome, inflammatory activation and tissue damage. Although this is an interesting idea at first glance, a large prospective study is needed to direct test our hypothesis.

Our study has some limitations. We had a limited sample size, and limited number of oxidative markers to establish a more detailed profile of oxidative disorder in SCA. The present investigation was exploratory and was focused on a particular population composed by children and adolescents. Regardless of such limitations, the robust multidimensional analysis adapted to relative limited sample size was able to reveal distinct profiles that could characterize the disease groups. In summary, our findings extend the current knowledge about SCA, highlighting more nuances of the systemic oxidative stress that characterizes this disease. We also propose a strategy to measure DOP to track disease activity, which could be useful in the clinical management of patients. Additionally, our observations suggest that hydroxyurea therapy may mitigate the degree of oxidative perturbation by activating coordinated mechanisms. A larger, prospective interventional study is warranted to findings on hydroxyurea described here.

## Supplementary information

Supplementary Information.

## Data Availability

The datasets generated during and/or analyzed during the current study are available from the corresponding author on reasonable request.

## References

[1] Engel ER, Howard AL, Ankus EJ, Rico JF (2020). Advances in sickle cell disease management. Adv. Pediatr..

[2] Hermann PB, Pianovski MA, Henneberg R, Nascimento AJ, Leonart MS (2016). Erythrocyte oxidative stress markers in children with sickle cell disease. J. Pediatr..

[3] Sabarense AP, Lima GO, Silva LM, Viana MB (2015). Characterization of mortality in children with sickle cell disease diagnosed through the Newborn Screening Program. J. Pediatr..

[4] de Almeida Oliveira, D. C. *et al.* Sickle cell disease retinopathy: characterization among pediatric and teenage patients from northeastern Brazil. *Rev Bras Hematol Hemoter* **36**, 340–344, doi:10.1016/j.bjhh.2014.07.012 (2014). 10.1016/j.bjhh.2014.07.012 PMC4318384 25305166

[5] Singh PC, Ballas SK (2015). Emerging drugs for sickle cell anemia. Expert Opin. Emerg. Drugs.

[6] Walters MC, Nienhuis AW, Vichinsky E (2002). Novel therapeutic approaches in sickle cell disease. Hematol. Am. Soc. Hematol. Educ. Program.

[7] Kato GJ, Steinberg MH, Gladwin MT (2017). Intravascular hemolysis and the pathophysiology of sickle cell disease. J. Clin. Invest..

[8] Potoka KP, Gladwin MT (2015). Vasculopathy and pulmonary hypertension in sickle cell disease. Am. J. Physiol. Lung. Cell Mol. Physiol..

[9] Kato GJ (2018). Sickle cell disease. Nat. Rev. Dis. Primers.

[10] Nur E (2011). Oxidative stress in sickle cell disease; pathophysiology and potential implications for disease management. Am. J. Hematol..

[11] Hierso R (2014). Effects of oxidative stress on red blood cell rheology in sickle cell patients. Br. J. Haematol..

[12] Yawn BP (2014). Management of sickle cell disease: summary of the 2014 evidence-based report by expert panel members. JAMA.

[13] Ataga KI (2017). Crizanlizumab for the prevention of pain crises in sickle cell disease. N. Engl. J. Med..

[14] Badat M, Davies J (2017). Gene therapy in a patient with sickle cell disease. N. Engl. J. Med..

[15] Nevitt SJ, Jones AP, Howard J (2017). Hydroxyurea (hydroxycarbamide) for sickle cell disease. Cochrane Database Syst. Rev..

[16] Atweh GF (2010). Hydroxyurea in sickle cell disease: what will it take to change practice?. Am. J. Hematol..

[17] Teixeira RS (2019). Higher values of triglycerides: HDL-cholesterol ratio hallmark disease severity in children and adolescents with sickle cell anemia. Braz. J. Med. Biol. Res..

[18] Teixeira RS (2017). Associations between endothelial dysfunction and clinical and laboratory parameters in children and adolescents with sickle cell anemia. PLoS ONE.

[19] Amaral EP (2016). N-acetyl-cysteine exhibits potent anti-mycobacterial activity in addition to its known anti-oxidative functions. BMC Microbiol..

[20] Barretto JR (2020). Heightened plasma levels of transforming growth factor beta (TGF-beta) and increased degree of systemic biochemical perturbation characterizes hepatic steatosis in overweight pediatric patients: a cross-sectional study. Nutrients.

[21] Vinhaes CL (2020). Newborns with Zika virus-associated microcephaly exhibit marked systemic inflammatory imbalance. J Infect Dis.

[22] Vinhaes CL (2019). Changes in inflammatory protein and lipid mediator profiles persist after antitubercular treatment of pulmonary and extrapulmonary tuberculosis: a prospective cohort study. Cytokine.

[23] Cruz LAB (2019). Chronic hepatitis B virus infection drives changes in systemic immune activation profile in patients coinfected with *Plasmodium vivax *malaria. PLoS Negl Trop Dis.

[24] Fernandes CD (2019). Host inflammatory biomarkers of disease severity in pediatric community-acquired pneumonia: a systematic review and meta-analysis. Open Forum Infect. Dis..

[25] Oliveira-de-Souza D (2019). Molecular degree of perturbation of plasma inflammatory markers associated with tuberculosis reveals distinct disease profiles between Indian and Chinese populations. Sci. Rep..

[26] Oliveira-de-Souza D (2020). Aging increases the systemic molecular degree of inflammatory perturbation in patients with tuberculosis. Scientific Reports.

[27] Vinhaes CL (2020). Chronic hepatitis B infection is associated with increased molecular degree of inflammatory perturbation in peripheral blood. Viruses.

[28] Vinhaes CL (2020). An inflammatory composite score predicts mycobacterial IRIS in people with HIV and severe lymphopenia: a prospective international cohort study. J. Infect. Dis..

[29] Voskou S, Aslan M, Fanis P, Phylactides M, Kleanthous M (2015). Oxidative stress in beta-thalassaemia and sickle cell disease. Redox. Biol..

[30] Hanson MS (2011). Methaemalbumin formation in sickle cell disease: effect on oxidative protein modification and HO-1 induction. Br. J. Haematol..

[31] Rees DC, Gibson JS (2012). Biomarkers in sickle cell disease. Br. J. Haematol..

[32] Damanhouri GA (2015). Clinical biomarkers in sickle cell disease. Saudi J Biol Sci.

[33] Amaral EP (2020). The interplay between systemic inflammation, oxidative stress, and tissue remodeling in tuberculosis. Antioxid. Redox Signal.

[34] Quintela-Carvalho G (2017). Heme drives oxidative stress-associated cell death in human neutrophils infected with *Leishmania infantum*. Front. Immunol..

[35] Andrade BB (2010). Heme impairs prostaglandin E2 and TGF-beta production by human mononuclear cells via Cu/Zn superoxide dismutase: insight into the pathogenesis of severe malaria. J. Immunol..

[36] Antwi-Boasiako C (2019). Oxidative profile of patients with sickle cell disease. Med. Sci..

[37] Loboda A (2008). Heme oxygenase-1 and the vascular bed: from molecular mechanisms to therapeutic opportunities. Antioxid. Redox Signal.

[38] Van Zwieten R, Verhoeven AJ, Roos D (2014). Inborn defects in the antioxidant systems of human red blood cells. Free Radic. Biol. Med..

[39] Schimmel M (2017). Inflammatory and endothelial markers during vaso-occlusive crisis and acute chest syndrome in sickle cell disease. Am. J. Hematol..

[40] Aslan M (2001). Oxygen radical inhibition of nitric oxide-dependent vascular function in sickle cell disease. Proc. Natl. Acad. Sci. USA.

[41] Nader E, Romana M, Connes P (2020). The red blood cell-inflammation vicious circle in sickle cell disease. Front. Immunol..

[42] Klings ES, Farber HW (2001). Role of free radicals in the pathogenesis of acute chest syndrome in sickle cell disease. Respir. Res..

[43] Nath KA (2005). Transgenic sickle mice are markedly sensitive to renal ischemia-reperfusion injury. Am. J. Pathol..

[44] Morris CR (2008). Erythrocyte glutamine depletion, altered redox environment, and pulmonary hypertension in sickle cell disease. Blood.

[45] Nur E (2010). Plasma levels of advanced glycation end products are associated with haemolysis-related organ complications in sickle cell patients. Br. J. Haematol..

[46] Steinberg MH (2010). The risks and benefits of long-term use of hydroxyurea in sickle cell anemia: A 17.5 year follow-up. Am. J. Hematol..

[47] Rigano P (2018). Real-life experience with hydroxyurea in sickle cell disease: a multicenter study in a cohort of patients with heterogeneous descent. Blood Cells Mol. Dis..

[48] Charache S (1995). Effect of hydroxyurea on the frequency of painful crises in sickle cell anemia. Investigators of the Multicenter Study of Hydroxyurea in Sickle Cell Anemia. N. Engl. J. Med..

[49] Wang WC (2011). Hydroxycarbamide in very young children with sickle-cell anaemia: a multicentre, randomised, controlled trial (BABY HUG). Lancet.

[50] Nader E (2018). Hydroxyurea therapy modulates sickle cell anemia red blood cell physiology: impact on RBC deformability, oxidative stress, nitrite levels and nitric oxide synthase signalling pathway. Nitric Oxide.

[51] Santana SS (2020). Hydroxyurea scavenges free radicals and induces the expression of antioxidant genes in human cell cultures treated with hemin. Front. Immunol..

