# Supplementary material for: Hydroxyurea treatment is associated with reduced degree of oxidative perturbation in children and adolescents with sickle cell anemia
Source: Sci Rep. 2020 Nov 4;10:18982. doi: 10.1038/s41598-020-76075-5 (PMC7642412; doi:10.1038/s41598-020-76075-5)
Supplement: Supplementary file 1 — Supplementary Information. [file 41598_2020_76075_MOESM1_ESM.docx]

Hydroxyurea Treatment is Associated with Reduced Degree of Oxidative Perturbation in Sickle Cell Anemia Children and Adolescents

Caian L. Vinhaes, Rozana S. Teixeira, Jay A. S. Monteiro-Júnior, Rafael Tibúrcio, Juan M. Cubillos-Angulo, María B. Arriaga, Adrielle G. Sabarin, Amâncio J. Souza, Jacqueline J. Silva, Isa M. Lyra, Ana Marice Ladeia and Bruno B. Andrade

**Supplementary Material**

**Table S1.** Characteristics of Study Participants

| Characteristic | Healthy Control (n=40) | Sickle Cell No HU  (n=27) | Sickle Cell HU  (n=20) | *P-value* |  |
| --- | --- | --- | --- | --- | --- |
| Age (year) | 11 (9-14.8) | 14 (11-16) | 12.5 (7.5-15) | 0.09 |  |
| Biological sex  Female  Male | 27 (67.5%)  13 (32.5%) | 10 (37%)  17 (63%) | 10 (50%)  10 (50%) | **0.004** |  |
| Ethinicity^#^  Non-White  White | 33 (85%)  6 (15%) | 25 (92.6%)  2 (7.4%) | 20 (100%)  0 | 0.1 |  |
|  |  |  |  |  |  |

Data represent median and interquartile range (IQR) for age or no. (%) and were compared using the Mann-Whitney *U* test or the Fisher’s exact test (categorical variables), respectively. ^#^Ethinicity data of one of the 40 controls participants was lost.

**Table S2.** Expression of Oxidative Stress Related Markers Between the Clinical Groups

| Parameter | Unit | Healthy Control (n=40) | Sickle Cell  Anemia (n=47) | *P-value* |  |
| --- | --- | --- | --- | --- | --- |
| HO-1 | ng/mL | 17.1  (14.9-21.2) | 93.9  (77.23-120.3) | **<0.001** |  |
| SOD protein | ng/mL | 159.4  (137.8-197) | 167.7  (142.0-208.9) | 0.58 |  |
| GSH | mmol/mL | 159.9  (142.3-196.3) | 162.4  (133.8-194.6) | 0.91 |  |
| Total oxidant status | µmol H_2_0_2_ Equiv./L | 2.1  (1.0-3.9) | 5.8  (3.6-8.5) | **<0.001** |  |
| Total antioxidant status | Trolox mM | 0.34  (0.26-0.54) | 0.28  (0.19-0.38) | **0.35** |  |
| MDA | mmol/mL | 2.3  (1.3-3.7) | 5.8  (3.7-8.6) | **<0.001** |  |
| LDH | ng/mL | 909  (778.5-1130) | 2124  (1752-2724) | **<0.001** |  |
| VEGF | pg/mL | 101.5  (69.2-167) | 183.4  (137.8-283.2) | 0.07 |  |
| sCD14 | µg/mL | 2.5  (1.8-3.2) | 3.98  (3.4-5.1) | **<0.001** |  |

Data represent median and interquartile range (IQR) and were compared using the Mann-Whitney *U* test. HO-1: Heme oxygenase 1. SOD: Superoxide Dismutase. GSH: Glutathione. MDA: Malondialdehyde. LDH: Lactate dehydrogenase. VEGF: Vascular endothelial growth factor.
